# A New Deep Hybrid Boosted and Ensemble Learning-Based Brain Tumor Analysis Using MRI

**DOI:** 10.3390/s22072726

**Published:** 2022-04-01

**Authors:** Mirza Mumtaz Zahoor, Shahzad Ahmad Qureshi, Sameena Bibi, Saddam Hussain Khan, Asifullah Khan, Usman Ghafoor, Muhammad Raheel Bhutta

**Affiliations:** 1 Department of Computer & Information Sciences (DCIS), Pakistan Institute of Engineering and Applied Sciences (PIEAS), Islamabad 45650, Pakistan; mumtazzahoor_18@pieas.edu.pk (M.M.Z.); drsaqureshi@pieas.edu.pk (S.A.Q.); hengrshkhan822@gmail.com (S.H.K.); asif@pieas.edu.pk (A.K.); 2 Pattern Recognition Lab, (DCIS), PIEAS, Islamabad 45650, Pakistan; 3 Faculty of Computer Science, Ibadat International University, Islamabad 54590, Pakistan; 4 Department of Mathematics, Air University, Islamabad 44000, Pakistan; sameenabibi@mail.au.edu.pk; 5 Department of Computer System Engineering, University of Engineering and Applied Science (UEAS), Swat 19060, Pakistan; 6 PIEAS Artificial Intelligence Center (PAIC), PIEAS, Islamabad 45650, Pakistan; 7 Department of Mechanical Engineering, Institute of Space Technology, Islamabad 44000, Pakistan; 8 School of Mechanical Engineering, Pusan National University, Busan 46241, Korea; 9 Department of Computer Science and Engineering, Sejong University, Seoul 05006, Korea

**Keywords:** brain tumor, analysis, detection, classification, hybrid learning, deep-boosted learning, ensemble learning, transfer learning, convolutional neural network

## Abstract

Brain tumor analysis is essential to the timely diagnosis and effective treatment of patients. Tumor analysis is challenging because of tumor morphology factors like size, location, texture, and heteromorphic appearance in medical images. In this regard, a novel two-phase deep learning-based framework is proposed to detect and categorize brain tumors in magnetic resonance images (MRIs). In the first phase, a novel deep-boosted features space and ensemble classifiers (DBFS-EC) scheme is proposed to effectively detect tumor MRI images from healthy individuals. The deep-boosted feature space is achieved through customized and well-performing deep convolutional neural networks (CNNs), and consequently, fed into the ensemble of machine learning (ML) classifiers. While in the second phase, a new hybrid features fusion-based brain-tumor classification approach is proposed, comprised of both static and dynamic features with an ML classifier to categorize different tumor types. The dynamic features are extracted from the proposed brain region-edge net (BRAIN-RENet) CNN, which is able to learn the heteromorphic and inconsistent behavior of various tumors. In contrast, the static features are extracted by using a histogram of gradients (HOG) feature descriptor. The effectiveness of the proposed two-phase brain tumor analysis framework is validated on two standard benchmark datasets, which were collected from Kaggle and Figshare and contain different types of tumors, including glioma, meningioma, pituitary, and normal images. Experimental results suggest that the proposed DBFS-EC detection scheme outperforms the standard and achieved accuracy (99.56%), precision (0.9991), recall (0.9899), F1-Score (0.9945), MCC (0.9892), and AUC-PR (0.9990). The classification scheme, based on the fusion of feature spaces of proposed BRAIN-RENet and HOG, outperform state-of-the-art methods significantly in terms of recall (0.9913), precision (0.9906), accuracy (99.20%), and F1-Score (0.9909) in the CE-MRI dataset.

## 1. Introduction

The brain is a complex and vital organ of the human body, controlling the nervous system. Irregular and uncontrolled growth of cells in the brain can cause a brain tumor. Brain tumors are usually categorized into primary and secondary tumors. The creation of brain tumors is not identifiable along with the growth rate, and brain tumors have the world’s highest mortality ratio of cancers. Primary brain tumors are devised in the brain tissues, whereas secondary tumors are produced in some other part of the body and shift to the brain through blood flow. Among the primary brain tumors, meningioma, glioma, and pituitary are harmful types of brain tumors and are most challenging for their early detection and effective treatment. Furthermore, these may lead to critical conditions if not addressed in a timely manner [1].

Early detection and classification of brain tumors with high prognosis accuracy is the most critical step for diagnosis and treatment to save a patient’s life. However, the manual analysis of brain MR images is laborious for radiologists and doctors to detect as is localizing the tumor and normal tissues and categorizing the tumors from medical images [2]. A computer-aided diagnosis (CADx) system is essential to overcome this problem. It needs to be implemented to relieve the workload and facilitate radiologists or doctors in medical images analysis. In the past, numerous researchers proposed several robust and accurate solutions to automate the brain tumor detection and classification task. 

Conventional machine learning (ML)-based approaches have been employed for brain tumor analysis. However, ML-based techniques entail manual feature extraction and classification and also are used on limited data. Deep learning (DL) has combined feature extraction and classification into a self-learning manner on a significant amount of labeled data, which considerably improved the performance. Moreover, CNN is a branch of DL, specially designed for image or two-dimensional (2D) data. It only takes datasets with minimal preprocessing and captures various features from MR images without human intervention [3]. Deep CNN models are largely used for brain tumor detection, classification, and segmentation. However, brain tumor analysis is highly challenging because of variable morphological structure, complex tumor appearance in an image, and nonlinear illumination effects which need an efficient DL-based brain tumor analysis system to strengthen the radiologist’s decision.

In this regard, we develop a deep-boosted hybrid learning-based approach to overcome these limitations by customizing the CNN models to exploit brain tumor-specific patterns from the brain MRI dataset. CNNs have shown admirable performance for identifying tumors from normal individuals and segregation of tumor types by using medical images. Moreover, deep feature boosting, ensemble learning, and ML classifiers help to improve performance considerably. Experimental results suggest that the proposed deep learning-based approaches would assist radiologists in diagnosing tumors and other irregularities from medical imaginings. The key contributions of the work are listed as follows.

1. An automated two-phase deep hybrid learning-based detection and classification (DHL-DC) framework is proposed for brain tumor analysis by using MRI images.
2. A novel deep-boosted features space, and ensemble classifiers (DBFS-EC)-based scheme is proposed to detect brain tumors. In this scheme, deep-boosted feature space is accomplished by using outperforming customized CNNs and provided to a majority voting-based ensemble of ML classifiers.
3. For the classification of brain tumor types, a new deep hybrid features space-based brain tumor classification approach is proposed. In the proposed technique, the dynamic features are obtained from the proposed novel brain region-edge net (BRAIN-RENet) and concatenated with a histogram of gradients (HOG) features to increase the feature space diversity and to improve the learning capacity of ML classifiers. Moreover, the proposed BRAIN-RENet carefully learns various tumors’ heteromorphic and inconsistent behavior.

The paper is arranged as follows. In Section 2 related work is discussed. Section 3 articulates the proposed methodology. Section 4 illustrates the experimental arrangements, and Section 5 is dedicated to results and discussion, and the conclusion is in Section 6.

## 2. Related Work

In medical image analysis, many research horizons are explored. These include various areas of medical imaging, such as detection, classification, and segmentation [4,5,6,7,8,9]. As cohorts build for brain tumor classification, there is a gap for novel approaches related to feature extraction by using limited and class-unbalanced MR images datasets of brain tumors and tumors from other parts of the human body [10,11]. Binary classifications are primitively explored in the literature to detect benign and malignant instances of the tumor. Kharrat et al. [12] explored the features of support vector machine (SVM) and genetic algorithm (GA) for brain tumor classification into three classes—normal, benign, and malignant. The proposed approach is only for binary classification. It is limited because it necessitates fresh training whenever there is a change in the image database. 

DL-based approaches performed better than conventional ML methods like Abdolmaleki et al. [13], who constructed a shallow neural network by using thirteen distinct features to distinguish benign and malignant tumors. These features were chosen based on the visual understanding of radiologists. The classification accuracy obtained by their proposed method was 91% and 94% for the malignant and descriptive tumors, respectively. Papageorgiou et al. [14] used fuzzy cognitive maps (FCM) to bifurcate the low- and high-grade gliomas. Their work achieved an accuracy of 93.22% for high-grade and 90.26% for low-grade brain tumors. Zacharaki et al. [15] proposed the feature selection scheme and then applied the conventional machine learning. They extracted the features like the shape of the tumor, tumor intensity, and invariant texture for this purpose. Feature selection and tumor classification are carried out by using SVM. Their work achieved the highest accuracy of 88% for low-grade and high-grade gliomas classification.

Khan, M.A. et al. [16] proposed a multi-model method for brain tumor classification by using DL. The proposed method is based on multiple steps, i.e., histogram equalization and discrete cosine transform, feature extraction by using pre-trained VGG16 and VGG19, and a correntropy-based learning method along with ELM for feature selection. Feature fusion is attained by using partial least square (PLS) and finally employing ELM for classification. Their method achieved accuracy of 97.8%, 96.9%, and 92.5% for BraTs2015, BraTs2017, and BraTs2018, correspondingly. M. Sarmad et al. [17] proposed edge detection-based fuzzy logic and a U-Net CNN-based brain tumor classification method. The proposed tumor segmentation system consists of image enhancement, fuzzy logic-based edge detection, and classification. They evaluate the proposed model by using accuracy, sensitivity, specificity, and a dice coefficient index. Raja, N.S.M. et al. [18] proposed image processing based on brain tumor classification by using thresholding and segmentation from 2D MRI slices. The tumor part is extracted by using the Modified Moth–Flame Optimization algorithm based on Kapur’s thresholding and a chosen segmentation technique by using benchmark datasets BRAIN-IX and TCIA-GBM. The study suggests that the proposed method performs slightly better on Flair modality images than the T2 modality.

Many researchers used the challenging benchmark dataset [19] consisting of MRI brain tumor scans with meningioma, gliomas, and pituitary-tumors. Cheng et al. [20] proposed a multi-phase brain tumor classification comprised of image dilation used as ROI and an augmentation of the tumor region in ring form. They evaluated their proposed model by using three different features and achieved 82.31% accuracy. In general, they improved their performance by using bag of the word (BOW) features, but the overall complexity of the model was increased. Sultan et al. [21] proposed a deep CNN-based brain tumor classification model and employed extensive data augmentation. They attained 96.13% accuracy for multi-class categorization. Ahmet and Muhammad [22] employed various deep CNN models for brain tumor analysis and achieved an accuracy of 97.2% with modified ResNet50 architecture. Khwaldeh et al. [23] employed multiple CNNs for brain MRI image classification and attained satisfactory accuracy. They attained a higher accuracy of 97.2% by using reformed pre-trained Alexnet CNN. In general, previously reported work aims to address the following points:

1. Most of the previously done works have been evaluated using accuracy on the validation dataset. However, precision, recall, and MCC are assessed for better performance evaluation on unbalanced datasets. Evaluation of such performance metrics is essential to measure the model’s generalization on the test dataset.
2. Previous work is largely restricted to either detection or classification of brain tumors. However, only the detection of tumors puts radiologists in an ambiguous situation due to insufficient details of the tumor type.
3. Largely normal individuals and tumors are classified in a single phase; this increased the overall complexity of models. Hence, isolating normal instances from tumor images for the classification phase may decrease the model’s complexity.

In this manner, the proposed two-phase brain tumor identification and classification framework can improve the diagnostics model’s performance by using standard performance assessment metrics like Accuracy, Precision, Recall, F-score, MCC, and AUC-PR.

## 3. Anticipated Methodology

**Figure 1:**
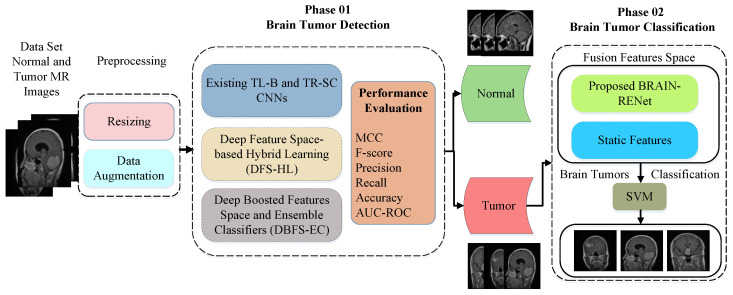
Overall description of the proposed two-phase brain tumor analysis framework.

The detailed architecture of the proposed DHL-DC framework is explained in this part. The proposed framework includes two phases. A deep-boosted features space and ensemble classifiers (DBFS-EC) method for brain tumor detection is proposed in the phase 01. In the phase 02, a hybrid features fusion-based brain tumor classification (HFF-BTC) model is proposed to classify brain tumor MR images detected from the first phase into three different classes: meningioma, glioma, and pituitary. MR images are categorized into different types by employing the proposed novel BRAIN-RENet based model. Feature space diversity is attained by composing a fusion feature space comprised of dynamic and static features. The proposed multi-stage brain tumor detection and classification framework is shown in Figure 1.

### 3.1. Preprocessing and Data Augmentation

In DL-based models, a deficient amount of data tends to model overt fit. Image augmentation is employed for efficient training and the improved generalization ability of models. Data augmentation techniques help to improve the performance of DL models [24,25]. This work employs four augmentation methods, shown in Table 1. Our dataset has MR images of different widths and heights, but it is recommended to resize them in the same height and width for obtaining optimum performance. In this proposed work, we resize the grayscale MR images into either 299 × 299 or 224 × 224 pixels.

### 3.2. Phase 01: Proposed Deep Learning-Based Brain Tumors Detection(DL-BTD) Scheme

**Figure 2:**
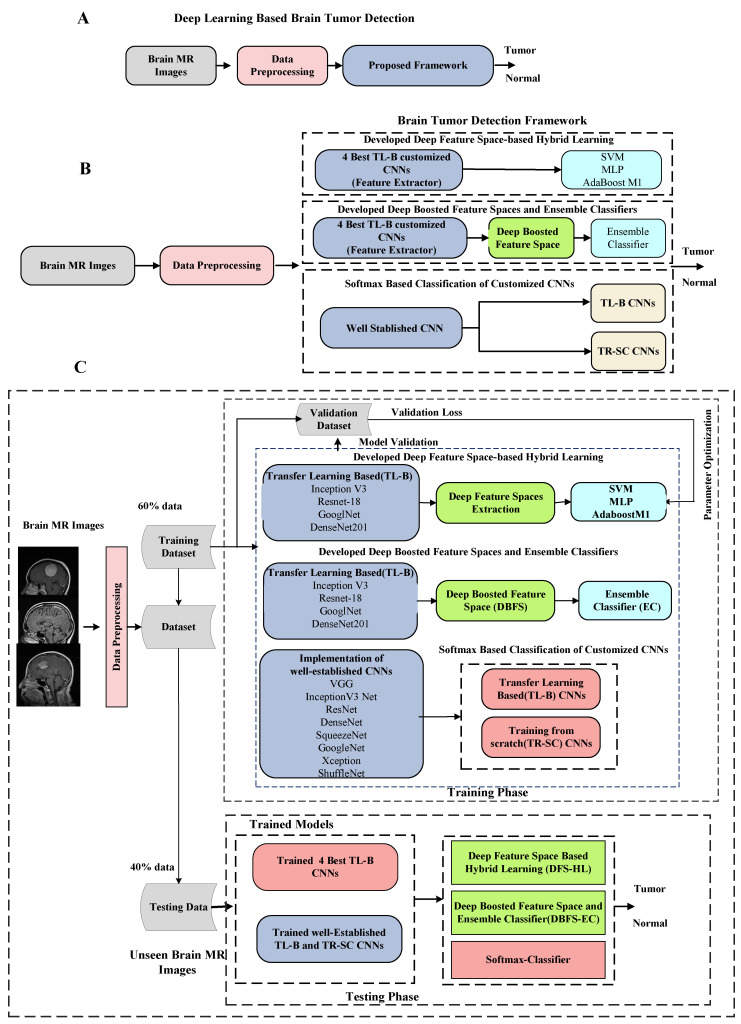
Overall detailed description of the proposed brain tumor analysis framework. (**A**) Block flow diagram. (**B**) Overall workflow overview. (**C**) Detailed block diagram of proposed deep learning-based brain tumor detection scheme.

We perform three distinct experimental approaches for brain tumor detection in the proposed scheme. It includes; (1) Softmax-based implementation of existing customized transfer learning-based (TL-B) and training from scratch (TR-SC) deep CNN models in an end-to-end mode to differentiate tumor and normal brain MRI images (2). Secondly, a deep feature space-based hybrid learning (DFS-HL) technique is designed. In DFS-HL, feature spaces of the four best TL-B fine-tuned models are fed into three ML classifiers to enhance the discrimination ability and generalization of the proposed scheme (3). In the third approach, we propose the DBFS-EC based framework to exploit the benefits of ensemble deep feature spaces and ensemble of ML classifiers. The block diagram of the DBFS-EC approach is shown in Figure 2.

#### 3.2.1. Implementation of Existing CNNs

In our proposed two-phase framework, both detection and classification models are trained independently. Initially, we employ two different methods in our proposed DL-BTD approach for Softmax-based classification by using TR-SC and TL-B fine-tuned deep CNN models. For TL-B and TR-SC, we employed ten well-known customized CNN models and trained them on the MRI image dataset. Employed CNN models include: i—VGG-16 [26], ii—VGG-19 [26], iii—SqueezeNet [27], iv—GoogleNet [28], v—ResNet-18 [29], vi—ResNet-50 [29], vii—XceptionNet [30], viii—InceptionV3 [31], ix—ShuffleNet [32], and x—DenseNet201 [33]. We trained all CNN architectures from scratch, and all layers of networks updated their weights consequently for TR-SC models. TL is employed to optimize well-established TL-B CNNs. We replace the input of the TL-B CNNs with a new one, which is the same as the size of the MRI images. The dimensions of the last fully connected layer of all deep CNNs are set the same as the number of the classes, i.e., two. The Softmax layer is employed to get the class-specific probabilities, and weights optimization is attained by using a backpropagation algorithm through minimizing the cross entropy-based loss function.

#### 3.2.2. Developed Deep Feature Spaces Based Hybrid Learning (DFS-HL)

Our suggested DFS-HL scheme selects four well-performing TL-B CNN models as feature extractors and fed feature vectors individually into three competitive ML classifiers. The MRI dataset used in this study is not enough for the training of the deep CNN model, and there are chances of overfitting. So we have incorporated TL by using the pre-trained weight of the CNN models on the ImageNet dataset. TL-B deep CNN models learn the most discriminative features efficiently. We employ three different ML classifiers for classification: SVM [34], MLP [35], and AdaBoostM1 [36]. In DFS-HL, deep CNNs minimize the empirical risk and reduce training error during optimal hyper-parameter selection [37]. In addition, ML classifiers aim to minimize the test error on the unseen data with a fixed distribution for the training set by exploiting the structural risk minimization principle and improving generalization.

#### 3.2.3. Developed Deep-Boosted Feature Space and Ensemble Classifier (DBFS-EC)

Ensemble learning aspires to performance improvement and encourages combining multiple feature vectors of various models into one rich information feature vector, and avoids the risk of using a feature vector extracted from a single model with unsatisfactory performance [38]. It can be applied to two schemes, i.e., feature ensemble and classifier ensemble, dependent on the fusion level. The features ensemble implicates concatenating feature sets provided to the ML classifier for the final result. In contrast, ensemble classifiers are based on voting strategy by integrating decisions from multiple classifiers. In the proposed DBFS-EC scheme, we use both features and classifiers ensemble techniques. We concatenate feature vectors of four best-performing TL-B CNN models to compose deep-boosted feature space (DBFS) shown in Equation (1) and an ensemble classifier (EC) by integrating all three classifiers on majority voting base for the final decision shown in Equation (2),

(1) $$f_{Boosted}=t_{c}\left(t_{CNN-1}\left(f\right)\left|\left|t_{CNN-2}\left(f\right)\right|\right|t_{CNN-3}\left(f\right)||t_{CNN-4}\left(f\right)\right)$$

(2) $$X\left(D\right)=\mathrm{mode}\left\{h_{1}\left(D\right),h_{2}\left(D\right),h_{3}\left(D\right)\right\}.$$

In Equation (1), $f_{Boosted}$ defines the boosted feature space and $t_{c}$ represents the feature spaces of all four CNNs individually. In Equation (2), X(D) denotes the final prediction of all three classifiers $h_{1}\left(D\right),h_{2}\left(D\right),\;\mathrm{and}\;h_{3}\left(D\right)$. The deep-boosted feature space of the four best CNNs and ensemble learning enhances the feature space diversity and discrimination power of the proposed DBFS-EC framework.

### 3.3. Phase 02: Proposed Brain Tumors Classification Framework

**Figure 3:**
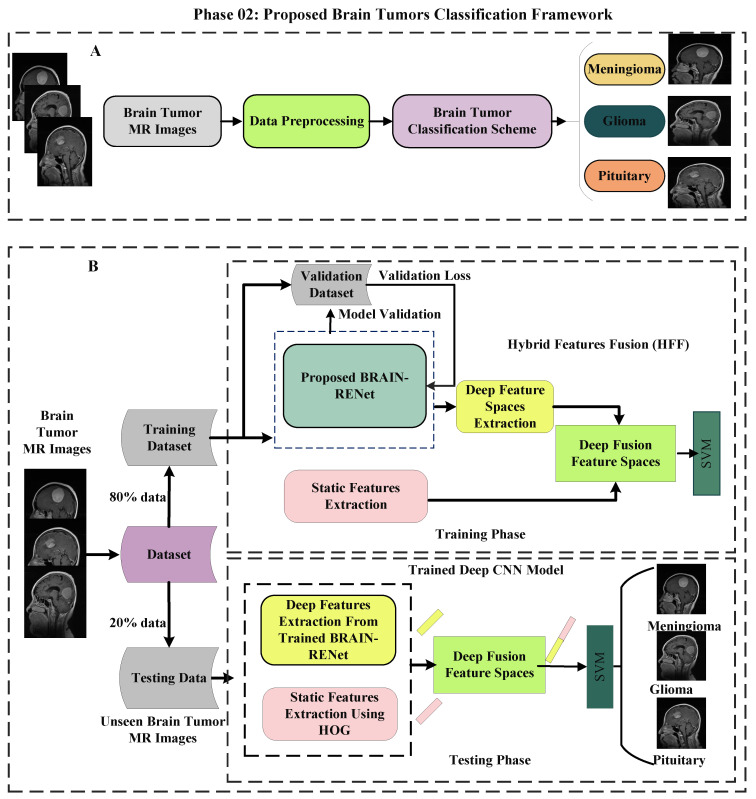
(**A**) Displays the concise details. (**B**) Demonstrates the comprehensive details of the proposed HFF-BTC model.

In the phase 02, a hybrid features fusion-based brain tumor classification (HFF-BTC) methodology for brain tumor categorization is proposed. Dynamic and static features are concatenated to enhance the feature space diversity, and an ML classifier is employed to improve the distinction power of the proposed approach. Deep features are extorted from the one layer before the last fully connected (FC) layer of the proposed novel BRAIN-RENet. Static features are extracted by using the HOG features descriptor. The proposed brain tumors classification framework is illustrated in Figure 3.

#### 3.3.1. Proposed Deep BRAIN-RENet

**Figure 4:**
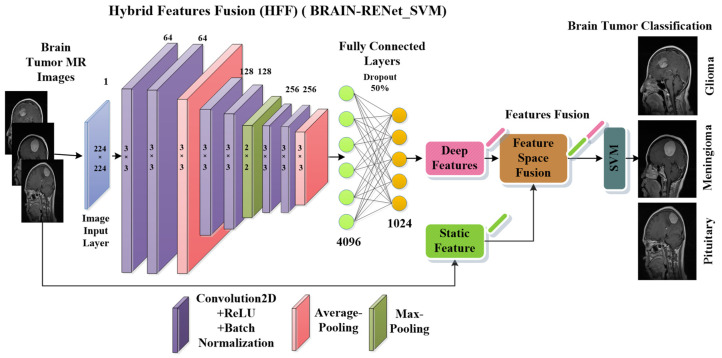
The proposed BRAIN-RENet deep CNN for brain tumor classification.

A novel brain tumor classification model using BRAIN-RENet is proposed in the phase 02 of the current study. A deep feature space that contains 1024 features is obtained from the one layer before the last fully connected layer of BRAIN-RENet, as shown in Figure 4. In the proposed BRAIN-RENet, systematic deployment of the region and edge operations exploit the region uniformity and edge-related features. We explored the benefits of systematic average- and max-pooling for distinguishing patterns of different brain tumors. Experiments prove that extracting edge- and region-based features enhances the proposed model’s performance. 

The proposed BRAIN-RENet contains six convolutional blocks. Every block is comprised of one convolutional layer, ReLU, and batch-normalization. The convolution layer extracts the tumor-specific features while ReLU act as an activation function. At the end of each block, average- and max-pooling operation is applied to learn region uniformity and boundary features of brain tumors as illustrated in Equations (4) and (5). Region and boundary-based operators are implemented systematically to capture patterns of different types of brain tumors. The detailed architecture of the proposed BRAIN-RENet is illustrated in Figure 4.

(3) $$W_{m,n}=\overset{r}{\underset{x=1}{\sum }}\overset{s}{\underset{y=1}{\sum }}W_{m+x-1,\;n+y-1\;}k_{a,b}$$

(4) $$W_{m,n}^{\mathrm{Avg}}=\frac{1}{T^{2}}\;\overset{t}{\underset{x=1}{\sum }}\overset{t}{\underset{y=1}{\sum }}W_{m+x-1,\;n+y-1\;}$$

(5) $$W_{m,n}^{\mathrm{Max}}={\mathrm{Max}}_{x=1\ldots t,\;y=1\ldots t}W_{m+x-1,\;n+y-1\;}$$

(6) $$Q=\overset{B}{\underset{b}{\sum }}\overset{C}{\underset{c}{\sum }}u_{d}W_{c}$$

The convolutional operation is used in Equation (3). The input feature-map has size M×N and is illustrated by w, and the filter of size r×s is denoted by k. The output feature map is illustrated by W, m, and n starting from 1 to (M−r+1) and (N−s+1), correspondingly. As illustrated in Equations (3)–(5), we determine the W^avg^ and W^max^ processes, denoted by W^Avg^ and W^Max^, respectively. In Equations (4) and (5), t denotes the average- and max-window sizes. In Equation (6), the output of the dense layer is defined by Q, which employ global operation on $W_{c}$; the output of the feature extraction phase. Neurons of FC layers are shown by ud. The FC layer at the ending obtains important features for the classification Equation (6). The CNN also contains a dropout layer to reduce overfitting. In brain MR images, various patterns show the intensity value variations in different image partitions. The smoothness of the region, changing texture, and edges form the pattern’s structure. MR images show the patterns of brain tumors, distinguished into different types of complexities and severity levels. 

In the proposed BRAIN-RENet, the systematic employment of edge- and region-based operations Equations (4) and (5), with a combination of convolutional operation Equation (3), facilitates the model for enhancing pattern-specific properties for brain tumor classification [39]. The advantages of the systematic employment of edge- and region-based operations in proposed BRAIN-RENet are as follows:

1. The proposed BRAIN-RENet improves imitating the image’s sharpness and smoothing dynamically, and can also fine tune the magnitude of smoothing and sharpening according to the spatial content of the image without human intervention.
2. Systematic employment of edge- and region-based operations after each convolutional block enhances the region homogeneity of different image segments.
3. The region operator smooths variations by applying average-pooling and suppresses the noise added during the MRI acquisition process. In contrast, the edge operator inspires CNNs for learning highly discriminative features by using a max-pooling operation.

#### 3.3.2. Hybrid Features Fusion-Based Brain Tumor Classification (HFF-BTC)

In our proposed hybrid features fusion-based brain tumor classification (HFF-BTC) model, we compose a hybrid feature space comprising static and dynamic features. Static features are extracted by using the HOG feature descriptor. Dynamic features are extracted by using the proposed BRAIN-RENet from the second last layer. Features fusion with hybrid learning exploited the advantages of empirical and structural risk minimization to enhance the performance of the brain tumor classification stage [37]. Deep CNNs contain strong learning ability and focus on reducing the empirical risk factor to minimize the training loss and to avoid overfitting [33]. The HOG feature descriptor counts the illustration of gradient orientation in local segments of an image. The HOG-descriptor emphasizes the shape or the structure of an object in images. [38]. The ML classifier SVM is used to minimize the structural risk factors, and hence improves generalization with the help of increased inter-class margins [39].

## 4. Experimental Setup

### 4.1. Dataset

**Figure 5:**
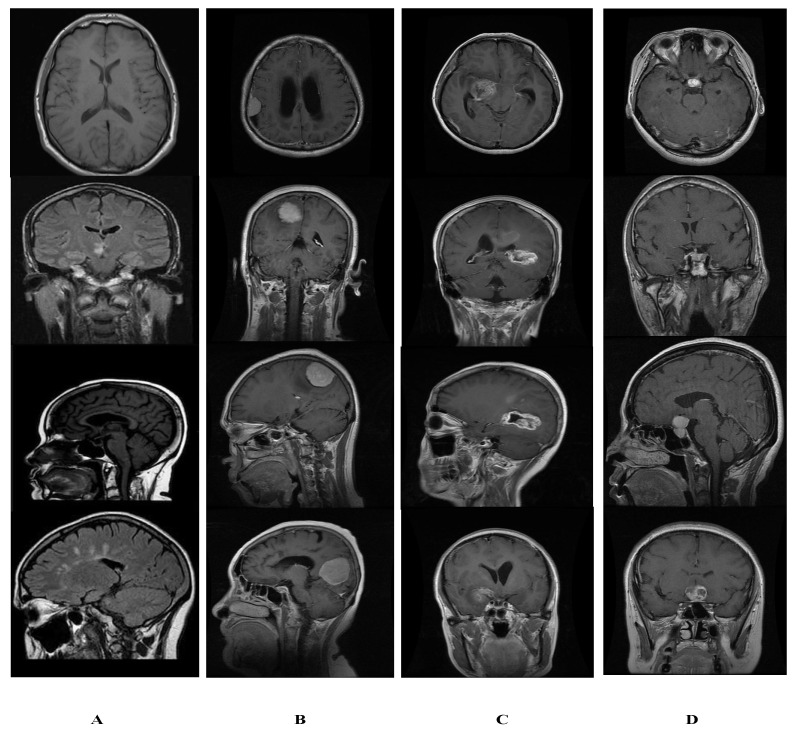
Sample image from dataset of normal and tumor images. (**A**) Normal. (**B**) Glioma. (**C**) Meningioma. (**D**) Pituitary.

We collect a brain tumor data set of normal and tumor images; normal images are collected from the open-source Kaggle website [40] and named as dataset1 (DS-1). Furthermore, tumor images are taken from a publicly available CE-MRI figshare [19], titled dataset2 (DS-2). We collected 5058 images containing 1994 healthy patients and 3064 tumor images; thus, the acquired dataset is imbalanced and called dataset3 (DS-3). In the phase 01, detection is performed on DS-3, which contains (5058) MR images, 3064 of which are tumor images, and 1994 of which are normal brain MR images. The classification stage categorizes tumor instances (3064) brain tumor MR images into different family classes, i.e., glioma, meningioma, and pituitary by using DS-2. Sample images of normal brain and tumors are shown in Figure 5.

### 4.2. Implementation Details

In the proposed work, we divide data into training and testing in the detection phase with a percentage of 60:40% for training and testing, correspondingly, and a percentage of 80:20% in the classification phase. Furthermore, the training data is again subdivided into train and validation sets for parameter optimization. Optimization of the model is attained by employing holdout cross-validation. We used stochastic gradient descent (SGD) [41] as an optimizer with a momentum of 0.95 in the training of CNNs. Training of deep models is run for 10 epochs, with a weight decay factor of 0.4, L2 regularization of 0.001, and a learning rate of 0.001. For efficient training, we have employed sixteen images for training one epoch. Cross-entropy loss is minimized by optimizing the CNN models for image classification. Softmax is employed as an activation function. All the CNN models are designed and simulations are performed by using MATLAB-2021a. Simulations were performed on Core-I, i7-7500 CPU by using a 2.90 GHz processor using CUDA₋enabled Nvidia^®^ GTX₋1060 Tesla. Our proposed model took almost 5–7 h, ~20–30 min/epoch for training. The experimental setup in both detection and classification approaches training was fixed for all networks in TL-B, TR-S, and proposed methods.

### 4.3. Assessment Metrics

The categorization capability of the proposed approaches is empirically assessed by using classification accuracy [42] (Acc.), recall (Rec.) [43], precision (Pre.) [43], F1-Score [44], Mathew Correlation Coefficient (MCC) [45], and PR Curve [46] which are expressed in Table 2, and Equations (7)–(11).

Acc = ((TN + TP)/(TP + TN+ FP + FN)) × 100(7)

Rec. = TP/(TP + FN)(8)

Pre. = TN/(TN + FP)(9)

F1-Score = (2 × (Pre. × Rec.))/(Pre. + Rec.)(10)

MCC = ((TP × TN) − (FP × FN))/√((TP + FP) × (FP + FN) × (TN + FP) × (TN+FN))(11)

## 5. Results and Discussion

A two-phase DL-based framework is designed for brain tumor analysis in this proposed work. In the phase 01, the detection of brain tumor individuals from normal instances is performed. In the phase 02, the classification of tumor images into further family classes is accomplished. Tumor detection alone is not completely beneficial for the successful curing process, hence, it is essential to classify tumors further into relevant classes for effective and efficient treatment. The empirical effectiveness of the proposed framework is evaluated by performing two experiments. In the first experiment, the brain tumor detection task is performed by assessing the performance of DL and DFS-HL-based models. In the phase 02, we evaluated the advantages of feature spaces fusion by combining dynamic-static feature spaces to discriminate patterns of different brain tumors. The suggested brain tumor analysis framework is validated on unseen data by using accuracy, sensitivity, precision, AUC-ROC, MCC, and F1-Score. The experimental results of dual stages are deliberated below.

### 5.1. Performance Evaluation of Tumor Screening Stage

In the proposed framework, initially, to categorize all samples into the tumor or healthy brain image, a DL-based DBFS-EC approach is proposed. Optimization of this stage results in minimum numbers of false positives for identifying tumors. The detection rate is enhanced by using three improvements in the detection phase. In the first step, we evaluate customized TR-SC and TL-B -based CNN models and determined that TL-B models perform better than TR-SC models ((0.75–8.4%) improvement in accuracy (Table 3)). The performance of TL-B CNN models is improved by replacing the Softmax layer with three ML classifiers after features extraction from fully connected layers of TL-B CNNs (0.24–0.65% improvement in accuracy (Table 4 and Table 5)). At last, our proposed novel DBFS-EC based detection approach further improves the performance ((1.39–9.05%) accuracy (Table 3 and Table 6)) of the brain tumor detection compared to existing customized CNN models.

#### 5.1.1. Distinction Competency of the Brain Tumor Detection Approach

Experimentation results for the proposed brain tumor detection scheme are obtained by using a combined dataset (DS-3). In the first experiment, we compare several customized TL-B and TR-SC deep CNN models by using an end-to-end way to obtain the tumor-related features. Performance assessment advocates that TL-B fine-tuned models learn the tumor-related feature better than the deep CNN models, trained from scratch on brain MR images. This is because of pre-trained weights that were learned from an extensive dataset named ImageNet [47]. 

As for the second experiment, we employ a hybrid learning-based approach by extracting the dominant features and exploiting the learning capability of deep CNNs with the strong discrimination power of ML classifiers. For this, we extract deep features spaces from the end layers of four best-performing TL-B deep CNNs (InceptionV3, ResNet18, GoogleNet, and DenseNet201), and fed them into competitive ML classifiers (SVM, MLP, and AdaBoostM1) The performance assessment based on accuracy, sensitivity, precision, and AUC-ROC, MCC, F1-Score, which are shown in Table 3, Table 4 and Table 5.

The deep features of DenseNet201 with all three ML classifiers perform better than extracted deep features of other pre-trained CNNs. However, the performance of the deep features extracted by using inceptionV3 fell shorter than features extracted from other pre-trained CNN networks on DS-3. The performance assessment is based on accuracy, recall, precision, F-score, and MCC (Table 4 and Table 5).

In the last experiment for the proposed DBFS-EC approach, the effectiveness of the deep-boosted ensemble learning is evaluated. A hybrid feature space is formed by concatenating all four selected feature spaces and ensemble classifier by using all three classifiers employed in experiment 2. Utilizing ensemble deep feature spaces from more than one TL-B deep CNNs is effective for ML classifiers. An ensemble of classifiers enhanced the overall performance of the proposed DBFS-EC approach for brain tumor detection.

Table 6 shows that DBFS-EC, an ensemble of deep feature spaces from top-4 TL-B CNN models and the ensemble of ML classifiers, achieves higher performance measures than the ensemble of deep features from top-4 pre-trained CNN models and ML classifiers individually (Table 4, Table 5 and Table 6). This is because ensemble learning employs feature spaces from four best-performing TL-B CNNs and concatenates them. Integrating these deep features results in a hybrid feature vector that increases the feature space diversity, and an ensemble of ML classifiers enhances the discrimination ability of the ML classifiers.

#### 5.1.2. ROC Curve-Based Performance Exploration

**Figure 6:**
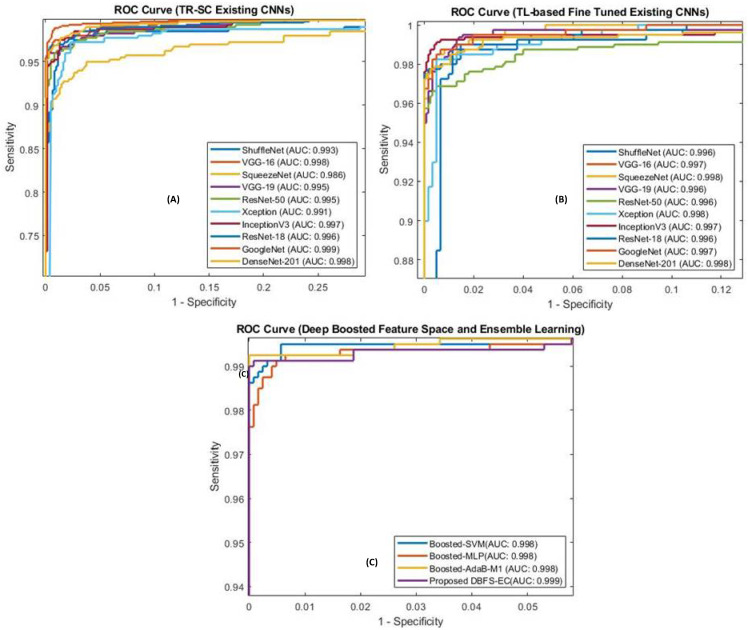
ROC curve for the existing CNNs, TR-SC based in (**A**), TL-B models in (**B**), and (**C**) represent proposed (DBFS-EC) framework.

Analyzing the ROC curve is crucial for achieving the optimum analytic threshold for a classifier. It pictorially shows the differentiation capacity of the classifier at possible threshold values. As shown in Figure 6, our proposed DBFS-EC scheme for the brain MRI dataset has improved performance (AUC_ROC: 0.999). ROC-based statistical analysis also highlights that the proposed approach attained high sensitivity.

### 5.2. Performance Analysis of Brain Tumor Classification Stage

Tumor classification is essential for designing effective treatment and diagnosis processes. Thus, brain MR images recognized as the tumor in the detection stage by using the proposed DBFS-EC are allocated to the brain tumor classification model for tumor categorization. In the classification phase, we have proposed a hybrid features fusion-based approach for tumor MR images categorization into particular classes, namely, meningioma, glioma, and pituitary. Dynamic and static features are concatenated to enhance the feature space diversity, and classification ability enhancement of the model is achieved by employing an ML classifier (SVM). Deep features are extracted by using the proposed novel BRAIN-RENet, and static features are extracted by using the HOG descriptor.

#### 5.2.1. Differentiation Proficiency of the Brain Tumor Classification Stage

The proposed model’s performance is assessed with the proposed BRAIN-RENet and several ML-based models. Table 7 shows the performance comparison of the proposed hybrid learning-based model containing fusion feature spaces with SVM, other HFF models, and the proposed BRAIN-RENet. Performance of the proposed HFF-BTC model is evaluated for standard metrics and attain recall (0.9913), precision (0.9906), accuracy (99.20%), and F1-Score (0.9909). This framework outperformed the existing techniques in recognizing the tumor in MRI images.

**Figure 7:**
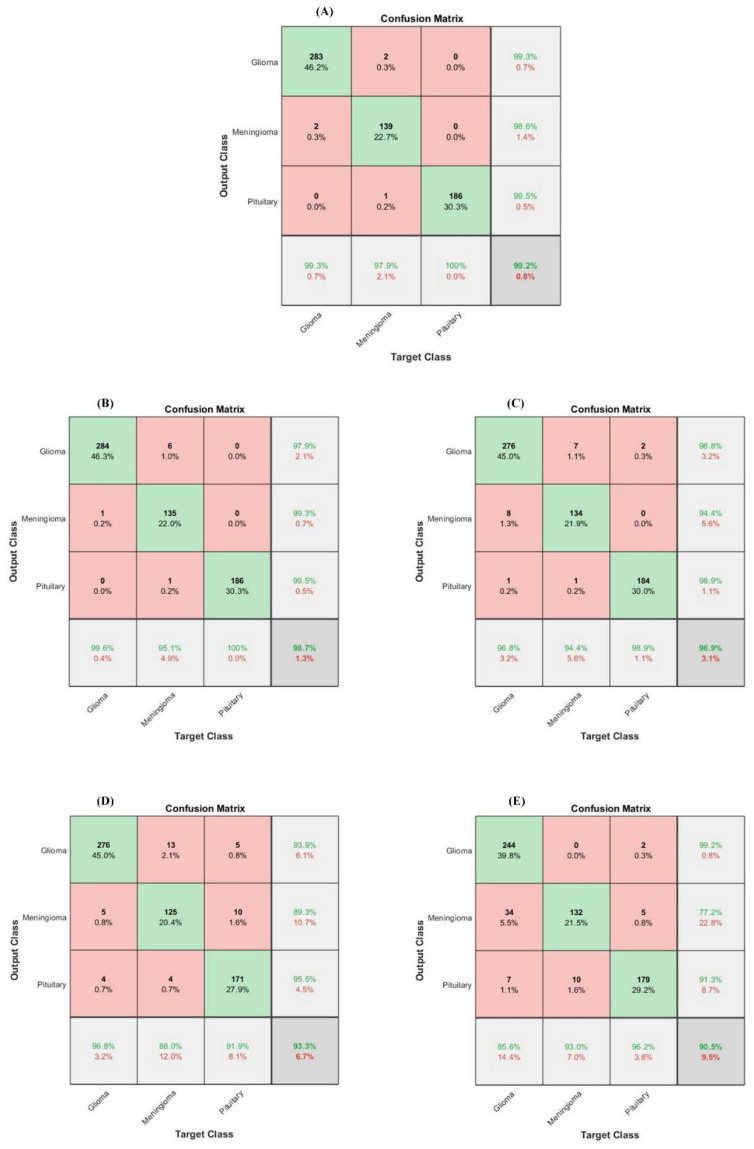
Confusion matrix-based performance comparison of proposed (**A**) HFF-BTC, (**B**) SVM poly. Ordr.2, (**C**) AdaboostM2, (**D**) Decision Tree, (**E**) Naïve Bayes.

In the brain tumor classification stage, we initially analyze the performance of our proposed HFF-BTC model with other existing hybrid learning-based models by using different ML classifiers, namely Naïve Bayes, Decision Tree, Ensemble (Adaboost-M2), and SVM with the linear, RBF, poly order-2 kernel. Confusion matrix-based performance comparison of proposed HFF-BTC with other existing ML methods is illustrated in Figure 7. We fed fusion feature space to several ML classifiers for performance evaluation. Table 7 and Figure 7 suggest that our proposed approach outperforms other models in terms of recall (0.9913), precision (0.9906), accuracy (99.2%), and F1-Score (0.9909). HFF and SVM with ploy order 3 learn and discriminate the tumor-specific patterns from MR images better than other classifiers with minimized false negatives.

**Figure 8:**
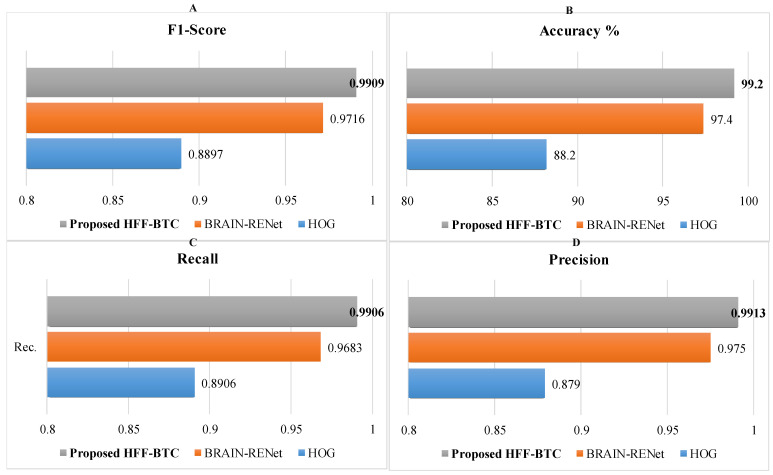
Performance comparison of HFF-BTC models in terms of (**A**) F1-score, (**B**) accuracy, (**C**) recall, and (**D**) precision.

Performance of the proposed HFF-BTC model using deep and static features individually and existing DL models are evaluated and demonstrated in Table 8 and Figure 8. Results show that the proposed HFF-BTC using SVM (with poly order 3) performs better than deep and static feature spaces separately and previously reported work. Cheng et al. [20] proposed multi-phase brain tumor classification comprises image dilation used as ROI and augmentation of the tumor region in ring form. They evaluated their proposed model by using three different features and achieved 91.28% accuracy. In general, they improved their performance by using bag of the word (BOW) features. Still, the overall complexity of the model was increased. Badža et al. [48] presented a CNN architecture for brain tumor classification and achieved an accuracy of 97.28%. The authors focused their work on auto-feature extraction by using a highly general model for brain tumor classification with good execution speed. Gumaei et al. [49] proposed a brain tumor classification approach by using hybrid feature-extraction methods with regularized extreme learning machines (RELM). Authors compute the covariance matrix to project these features in a new significant feature set and employ RELM for classification. Authors improved classification accuracy from 91.51% to 94.23% for the experiment of random holdout technique. Díaz Pernas et al. [49] proposed a fully automatic brain tumor classification and segmentation model by using a deep CNN based on a multiscale approach. They achieved an accuracy of 97.3% on a benchmark CE-MRI dataset. The proposed method achieves performance metrics of recall (0.9906), precision (0.9913), accuracy (99.2%), and F1-Score (0.9909). This improvement in the performance of the proposed HFF-BTC is attained by employing two techniques. First, we concatenate the dynamic and static feature spaces and then use SVM as a classifier. Systematic usage of max and average pooling in the proposed BRAIN-RENet enabled the model in identifying fine-grained details and features high discrimination in MRI images. In addition, the concentration of features enhances the feature space diversity, the inter-class association is maximized, and SVM helps in structural risk minimization

#### 5.2.2. Features Space Visualization

**Figure 9:**
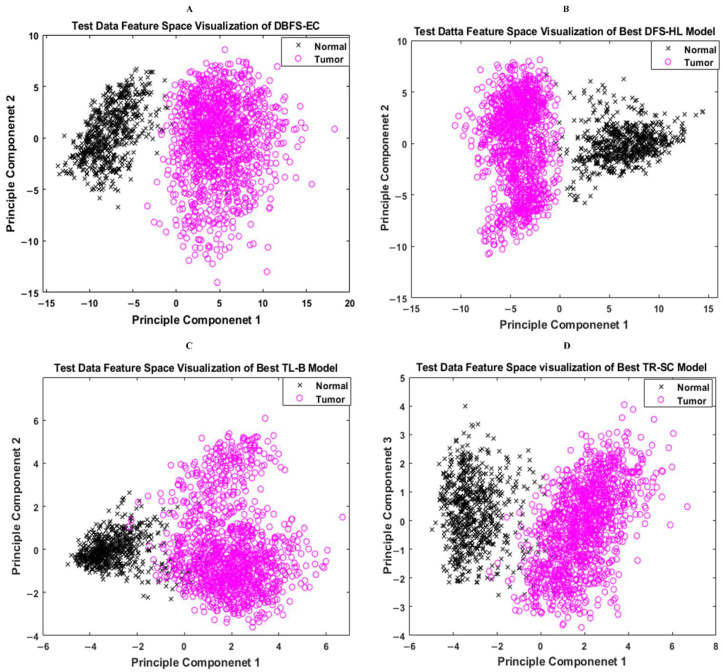
Feature space visualization of the proposed DBFS-EC framework (**A**), DFS-HL (**B**), well-performing TL-B (**C**), and TR-SC (**D**) models.

**Figure 10:**
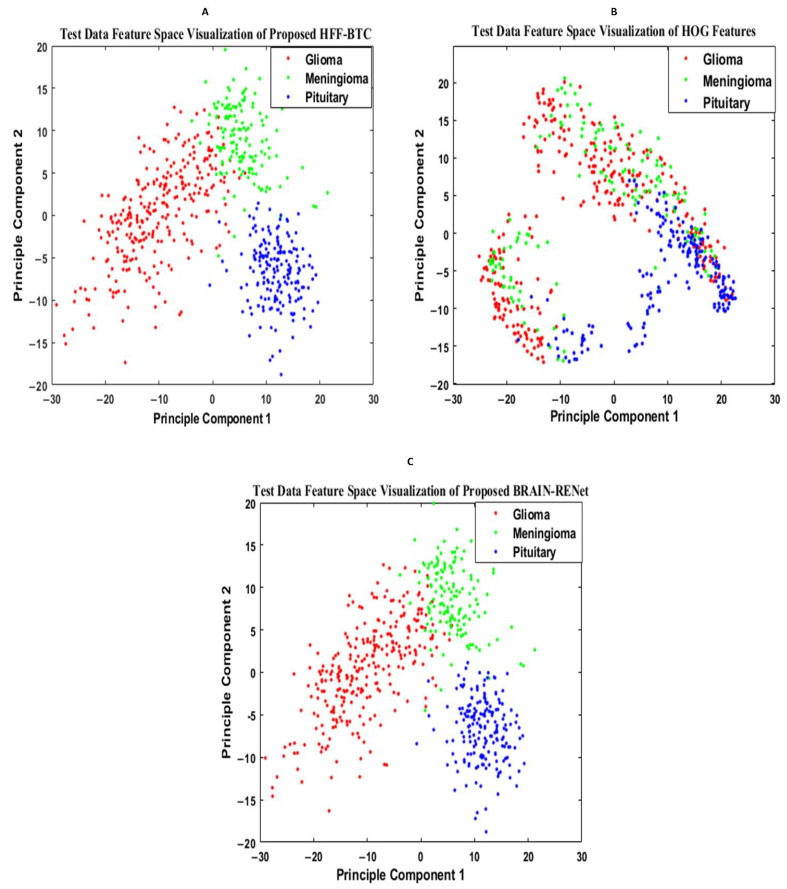
Feature space visualization of the proposed HFF-BTC framework (**A**), HOG descriptor (**B**), and proposed BRAIN-RENet (**C**).

Features space diversity attained by the proposed DBFS-EC and DFS-HL is assessed to elucidate the tumor detection and classification process. In general, the discrimination ability of the model is dependent on the associated properties of the features set. Distinguishing the class features strengthens the learning ability of the model and improves robustness on a wide-ranging set of instances. The proposed DBFS-EC framework boosted the feature space diversity and improved brain tumor recognition and categorization. The 2-D scatter plot of principal components (PC) and their divergence attained by proposed DBFS-EC with the comparison of best-performing TR-SC and TL-B deep CNNs on test data are shown in Figure 9 and by HFF-BTC compared with HOG and BRAIN-RENet in Figure 10.

## 6. Conclusions

An efficient brain tumor diagnosis system is necessary for the early treatment of the patient. In this regard, a new two-phase brain tumor detection and classification framework is proposed to improve brain tumor diagnosis and reduce computational complexity. In the detection phase, we proposed a novel DBFS-EC approach for differentiating brain tumor instances from normal individuals with fewer false negatives, and performance is compared with the existing DL techniques. Experimental results demonstrate that the proposed DBFS-EC outperformed other models by achieving better accuracy (99.56%), recall (0.9899), precision (0.9991), F1-Score (0.9945), and MCC (0.9892). In the tumor classification phase, an FSF-HL technique is proposed, which is based on novel brain-RENet model and benefits from feature space fusion and ML. The feature space fusion exploits region uniformity, edge-related, and static features and then provides to ML to improve the model generalization by reducing structure risk minimization. The proposed technique achieved recall (0.9906), precision (0.9913), accuracy (99.20%), and F1-score (0.9909) for brain tumor classification on a benchmark dataset. The two-phase framework is expected to assist clinicians in decision-making in clinical practice and will be helpful for radiologists in brain tumor diagnosis. In the future, we will appraise our proposed framework’s performance and a more optimized one on other large medical image datasets to improve efficacy and reliability for real-time detection and classification. In this regard, we will focus on augmenting the training sets by generating synthetic examples using the generative adversarial network.

## Figures and Tables

**Table 1:** Augmentation methods.

Method	Parameters
Image-Rotation	0 to 360 degree
Image-Sharing	−0.05, +0.05
Image-Scaling	0.5–1 limit
Image Reflection	±1 in the right–left direction

**Table 2:** Assessment Metric Details.

Metric	Description
Precision (Pre.)	The fraction of correctly detected class to an actual class
Recall (Rec)	The proportion of correctly identified class and actual negative class
Accuracy (Acc.)	% of the total number of correct detection
MCC	Matthews correlation coefficient
F1-Score	The harmonic mean of Pre. and Rec.
TP	Truly positive prediction
TN	Truly negative prediction
FP	Falsely positive prediction
FN	Falsely negative prediction

**Table 3:** Softmax probabilistic-based employment of custom-made CNN models 60:40% data portioning (training: testing).

Model	Training Scheme									
	Transfer Learning-Based (TL-B)					Training from Scratch (TR-SC)				
	Acc. %	Rec.	Pre.	F1-Score	MCC	Acc. %	Rec.	Pre.	F1-Score	MCC
ShuffleNet	98.52	0.9824	0.9868	0.9846	0.9694	90.51	0.9849	0.8702	0.9241	0.8455
VGG-16	98.76	0.9837	0.9901	0.9869	0.9739	94.07	0.9899	0.9155	0.9512	0.9016
SqueezeNet	98.91	0.9849	0.9917	0.9883	0.9768	95.36	0.9498	0.9556	0.9527	0.9058
VGG-19	98.22	0.9949	0.9744	0.9846	0.9691	96.54	0.9799	0.9569	0.9683	0.9362
ResNet-50	98.42	0.9649	0.9966	0.9805	0.9621	97.53	0.9448	0.9948	0.9692	0.9412
Xception	98.81	0.9824	0.9917	0.9807	0.9743	97.23	0.9599	0.9801	0.9698	0.9405
Inception-V3	98.52	0.9924	0.9806	0.9856	0.9730	97.63	0.9573	0.9882	0.9725	0.9464
Resnet-18	98.91	0.9774	0.9966	0.9869	0.9744	97.43	0.9812	0.9701	0.9756	0.9511
GoogleNet	98.52	0.9924	0.9806	0.9856	0.9731	97.53	0.9937	0.9643	0.9788	0.9575
DenseNet-201	98.86	0.9724	0.9991	0.9856	0.9720	98.17	0.9636	0.9932	0.9782	0.9576

**Table 4:** Performance comparison of Softmax probabilistic-based and deep feature extracted from custom-made TL-B CNNs with SVM-based classification of four best-performing TL-B CNN models selected for proposed DFS-BTD framework. 60:40% data portioning (training: testing).

Model	DFS-HL Scheme									
	Transfer Learning-Based (TL-B) Softmax Based Classification					4 Best Performing Transfer Learning-Based (TL-B) with SVM				
	Acc. %	Rec.	Pre.	F1-Score	MCC	Acc. %	Rec.	Pre.	F1-Score	MCC
Inception-V3	98.52	0.9924	0.9806	0.9856	0.973	99.01	0.9824	0.9950	0.9887	0.9776
Resnet-18	98.91	0.9774	0.9966	0.9869	0.9744	99.16	0.9799	0.9991	0.9894	0.9793
GoogleNet	98.52	0.9924	0.9806	0.9856	0.9731	99.11	0.9849	0.995	0.9899	0.9801
DenseNet-201	98.86	0.9724	0.9991	0.9856	0.9720	99.06	0.9887	0.9918	0.9902	0.9806

**Table 5:** Performance comparison of features extracted from custom-made TL-B CNN models with MLP- and AdaBoostM1-based classification of four best-performing TL-B CNN models selected for the proposed DFS-BTD framework. 60:40% data portioning (training: testing).

Model	DFS-HL Scheme									
	4 Best Performing Transfer Learning-Based (TL-B) with MLP					4 Best Performing Transfer Learning-Based (TL-B) with AdaBoostM1				
	Acc. %	Rec.	Pre.	F1-Score	MCC	Acc. %	Rec.	Pre.	F1-Score	MCC
Inception-V3	99.31	0.9824	1.0000	0.9911	0.9826	99.06	0.9899	0.9910	0.9905	0.9810
Resnet-18	99.26	0.9912	0.9934	0.9923	0.9847	99.41	0.9912	0.9959	0.9935	0.9872
GoogleNet	99.36	0.9874	0.9975	0.9924	0.9851	99.11	0.9824	0.9966	0.9895	0.9793
DenseNet-201	99.41	0.9862	0.9991	0.9926	0.9855	99.46	0.9874	0.9991	0.9932	0.9867

**Table 6:** Deep boosted feature space and ensemble classification (DBFS-EC) 60:40% data portioning (training: testing).

Classifiers	Deep Hybrid Boosted Feature Space				
	Acc. %	Rec.	Pre.	F1-Score	MCC
SVM	99.41	0.9924	0.9950	0.9937	0.9876
MLP	99.41	0.9974	0.9918	0.9940	0.9888
AdaboostM1	99.46	0.9862	1.0000	0.9941	0.9863
Proposed DBFS-EC	99.56	0.9899	0.9991	0.9945	0.9892

**Table 7:** Performance comparison of proposed HFF-BTC with existing ML models.

Classifiers	Parameters	HFF-HL			
		Rec.	Pre.	Acc. %	F1-Score
Naïve Nayes	Gaussian Kernel	0.9160	0.8923	90.5	0.9039
Decision Tree	-	0.9223	0.9280	93.3	0.9251
Ensemble	AdaboostM2	0.9670	0.9670	96.9	0.9670
SVM	Linear kernel	0.9743	0.9616	96.9	0.9679
	Poly. Order 2	0.9823	0.9890	98.7	0.9856
	RBF	0.9883	0.9866	98.9	0.9874
Proposed Framework (HFF-BTC)	Dynamic + Static-SVM	0.9906	0.9913	99.2	0.9909

**Table 8:** Performance evaluation of proposed HFF-BTC with state-of-the-art models.

Method	The Proposed Classification Setup			
	Rec.	Pre.	Acc. %	F1-Score
Cheng et al. [20]	0.8105	0.9201	91.28	-
Badža et al. [48]	0.9782	0.9715	97.28	0.9747
Gumaei et al. [49]	-	-	94.23	-
Díaz Pernas et al. [50]	-	-	97.30	-
Proposed BRAIN-RENet-SVM	0.9683	0.9750	97.40	0.9716
HOG-SVM	0.8906	0.8790	87.20	0.8897
Proposed HFF-BTC	0.9906	0.9913	99.20	0.9909

## Data Availability

Data are available in publicly accessible repositories which are described in Section 4.1.
